# Oral Treatment With Bisphosphonates of Osteoporosis Does Not Increase the Risk of Severe Gastrointestinal Side Effects: A Meta-Analysis of Randomized Controlled Trials

**DOI:** 10.3389/fendo.2020.573976

**Published:** 2020-11-10

**Authors:** Zsuzsa Réka Dömötör, Nóra Vörhendi, Lilla Hanák, Péter Hegyi, Szabolcs Kiss, Endre Csiki, Lajos Szakó, Andrea Párniczky, Bálint Erőss

**Affiliations:** ^1^ Faculty of Medicine, University of Medicine, Pharmacy, Science and Technology of Targu Mures, Targu Mures, Romania; ^2^ Institute for Translational Medicine, University of Pécs, Medical School, Pécs, Hungary; ^3^ Doctoral School of Clinical Medicine, University of Szeged, Szeged, Hungary

**Keywords:** bisphosphonate, drug safety, gastrointestinal adverse event, gastrointestinal side effect, meta-analysis

## Abstract

**Introduction:**

Bisphosphonates (BPs) are first-line therapy for osteoporosis. Adherence is usually low in chronic, asymptomatic diseases, but gastrointestinal (GI) side-effects can also contribute to low adherence in BP therapy and may necessitate a review by a gastroenterologist with or without gastroscopy.

**Aims:**

Our meta-analysis aims to determine the risk of severe GI adverse events due to oral BP therapy in osteoporotic patients.

**Methods:**

A systematic search was conducted in three databases up to September 2020 for randomized controlled trials (RCTs) detailing GI adverse events in adults with osteoporosis on BP compared to placebo. Risk ratios (RRs) with 95% confidence intervals (CI) were calculated for non-severe and severe adverse events indicating endoscopic procedure with the random-effects model. Statistical heterogeneity was assessed using chi^2^ and I^2^ statistics.

**Results:**

Forty-two RCTs with 39,047 patients with 9,999 non-severe and 1,503 severe GI adverse events were included. The incidence of non-severe and severe adverse events ranged between 0.3–54.9 and 0–10.3%, respectively. There was no difference between BP and control groups in terms of the risk of non-severe or severe side effects: RR=1.05 (CI: 0.98–1.12), I^2^ = 48.1%, and RR=1.01 (CI: 0.92–1.12), I^2^ = 0.0%, respectively. Subgroup analysis of the most commonly used BP, once-weekly alendronate 70 mg, revealed an association between bisphosphonates and the risk of non-severe GI adverse events, RR=1.16 (CI: 1.00–1.36), I^2^ = 40.7%, while the risk of severe GI side effects was not increased in this subgroup, RR=1.20 (CI: 0.83–1.74), I^2^ = 0.0%.

**Conclusion:**

Our results show that bisphosphonates do not increase the risk of severe GI adverse events. However, the marked variability of the screening for side effects in the included studies, and the fact that in most of the studies GI diseases were exclusion criteria limits the strenght of evidence of our results. The conclusions drawn from the meta-analysis are therefore restricted to selected populations, and the results must be interpreted with caution.

## Introduction

Osteoporosis is a systemic bone disease with low bone mineral density and poor bone microarchitecture which leads to an increased risk of fracture (1). According to the most recent Osteoporosis Guideline, oral bisphosphonates (BPs) are one of the most commonly used therapeutic agents in patients with osteoporosis (2). Adherence is usually low in chronic, asymptomatic diseases, but gastrointestinal (GI) side-effects can also contribute to low adherence in BP therapy and may necessitate a review by a gastroenterologist with or without gastroscopy (3, 4). A cross-sectional patient survey showed that these GI side effects account for 40% of all discontinuation (5). Most commonly these are reported in the foregut, including heartburn, nausea, vomiting, epigastric pain, esophagitis, gastric ulcer, dyspepsia, and GI bleeding (6).

While the efficacy of BPs is out of debate, previous systematic reviews and meta-analysis investigating the tolerability of bisphosphonates did not determine the risk of severe and non-severe GI side effects of oral bisphosphonates.

There have been studies investigating the bisphosphonates-caused mucosal damage of the upper GI tract since it became an established drug in the treatment of osteoporosis (7–9).

None of the previous meta-analyses in this topic focused on the risks of severe GI side effects. We aimed to differentiate between the mild and severe side effects and determine the risks of these side effects in case of all commonly used oral bisphosphonates for osteoporosis.

## Methods

### Protocol

Our meta-analysis and systematic review is reported using the Preferred Reporting Items for Systematic Review and Meta-Analysis Protocols (PRISMA-P) (10). The project was registered in October 2019 on PROSPERO the registration number is CRD42020147522.

### Eligibility Criteria

Our scientific question, using the population-intervention-control-outcomes (PICO) framework was: (P) adult patients with primary osteoporosis, (I) oral bisphosphonates, (C) placebo or vitamin D or calcium, but no other medication for osteoporosis, and (O) severe and non-severe GI adverse events. Articles were included if they provided relevant information about any drug-induced GI adverse event. Only full-text articles and randomized controlled trials (RCTs) were included.

### Search Strategy

A systematic search was conducted in 3 databases, MEDLINE (via PubMed), Embase, and Cochrane Central Register of Controlled Trials from inception to 6^th^ September 2020. Keywords for the computer-aided search were ((diphosphonate OR bisphosphonate OR etidron* OR clodron* OR tiludron* OR pamidron* OR neridron* OR olpadron* OR alendron* OR ibandron* OR risedron* OR zoledron*) AND (gastrointestinal OR digestive OR “alimentary tract” OR esophageal OR esophagus OR oesophageal OR oesophagus OR gastric OR stomach OR antrum OR antral OR pylorus OR pyloric OR gastroduodenal OR duodenal OR duodenum OR bowel OR intestine OR intestinal OR colon OR colonic OR viscus OR visceral OR abdomen OR abdominal)), with the “Human” filter applied, but without other restrictions to language or other features.

### Study Selection

Records were managed by EndNote X9 (Clarivate Analytics, Philadelphia, PA, USA). After the exclusion of duplicates, the remaining records were screened by title, abstract, and full-text independently by two review authors (ZRD, NV). Additional articles were manually searched and identified from the reference lists of eligible primary studies. Disagreements were resolved by consensus or by the involvement of the senior review author (BE).

### Data Extraction

Numeric data were extracted by two review authors (ZRD, NV) and manually populated onto a purpose-designed Excel 2019 sheet (Office 365, Microsoft, Redmond, WA, USA). Data were collected from each paper on the year of publication, study design, country, the number of randomized patients, and baseline patient characteristics (age, sex, race, history of GI, body mass index, tobacco, alcohol, and caffeine usage in both groups). Most importantly, data were collected on the non-severe and severe GI adverse events. To ensure that results of the included studies were uniformly assessed as intention-to-treat protocol, in cases of per-protocol analyses the missing data were imputed, missing subjects were regarded as not having adverse events. Adverse events reported in the original studies were categorized by the review authors following the U.S. Food and Drug Administration criteria (11), detailed in **Supplementary Table 1**. Data on type of the bisphosphonate used as treatment and the control treatment, dosage, duration, route, and schedule of administration, follow-up period were also extracted. Disagreements were resolved by consensus or by the involvement of the senior reviewer (BE).

### Statistical Analysis

Risk ratios (RRs) with 95% confidence intervals (CI) were calculated for non-severe and severe adverse events with the random-effect model by DerSimonian-Laird (12). Subgroup analyses were performed for the different bisphosphonates (alendronate, risedronate, etidronate, pamidronate, ibandronate), the different dosage of the bisphosphonates and the duration of administration. Statistical heterogeneity was assessed using chi^2^ and I^2^ statistics. Statistical heterogeneity was assessed using Cochrane’s Q and the I^2^ statistics. According to the Cochrane Handbook for Systematic Reviews of Interventions (13), heterogeneity could be interpreted as moderate between 30 and 60%, as substantial between 50 and 90% and as considerable above 75%. The presence of publication bias was assessed by visual inspection of the funnel plots and Egger’s test (14), and the effect of publication bias was evaluated by the trim-and-fill method (15). A significant test result from Egger’s test (p<0.1) indicates the presence of bias. We also performed Trial Sequential Analysis (TSA) for the primary outcomes to evaluate whether further randomized trials are futile to show or discard the anticipated intervention effect. Statistical analyses were performed with Stata 16 (Stata Corp, College Station, TX, USA) and trial sequential analysis program version 0.9 beta (available from www.ctu.dk/tsa).

### Risk of Bias Assessment

The quality assessment was done at the study level and then summarized. We used the revised Cochrane Collaboration’s risk-of-bias tool for randomized trials (16) for methodological quality assessment of the individual studies included in our meta-analysis. The risk of bias was assessed independently by three investigators (ZRD, NV, EC). Disagreements were resolved by consensus and the involvement of the corresponding author.

### Assessment of the Grade of Evidence

The GRADE approach was used to assess the certainty of evidence regarding the outcomes. GRADE stands for Grades of Recommendation Assessment, Development, and Evaluation (17).

GRADE was assessed independently by two investigators (ZRD, EC). Disagreements were resolved by consensus and with the involvement of the corresponding author.

## Results

### Results of the Selection Process

**Figure 1:**
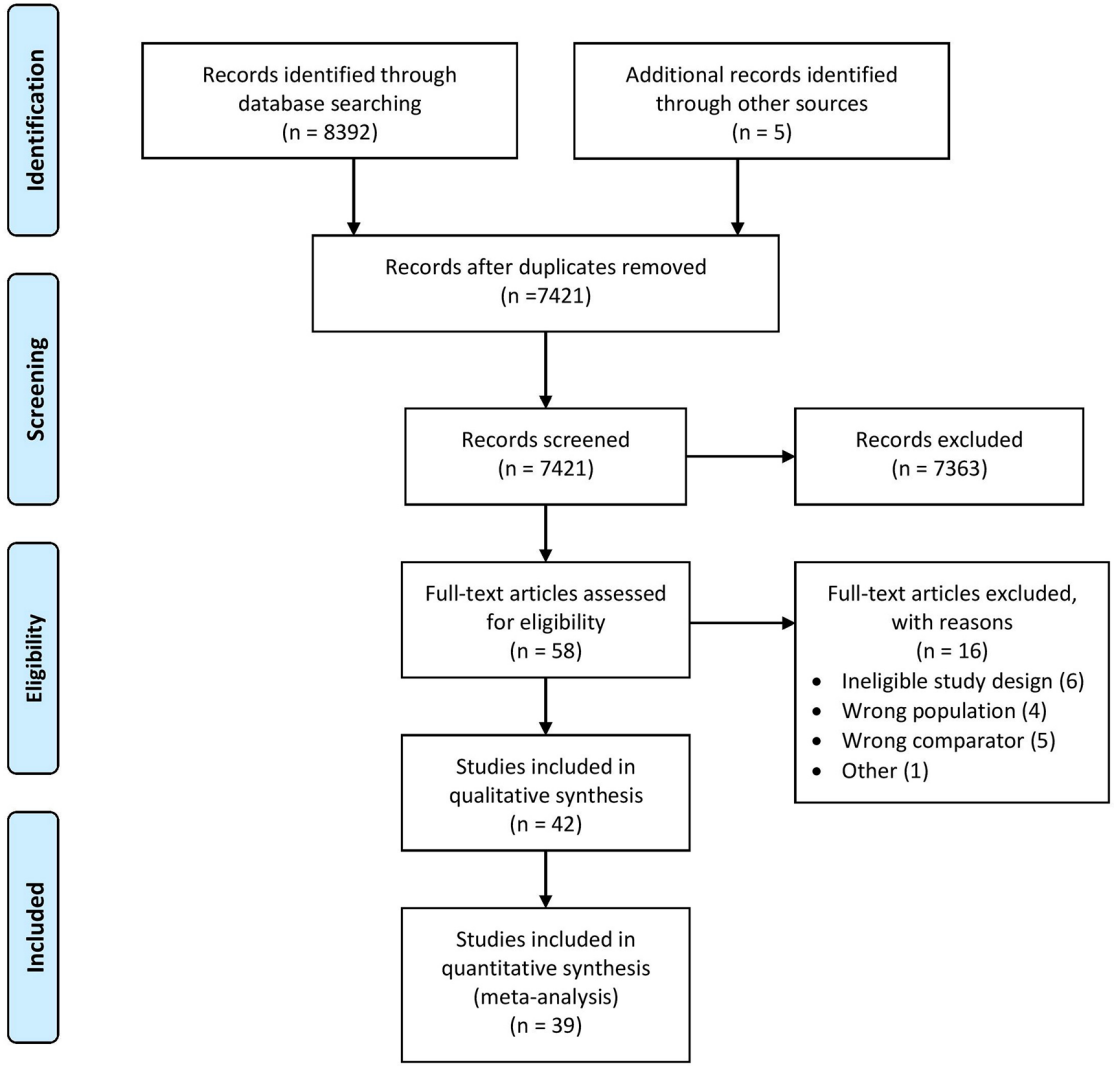
PRISMA flowchart.

Our search strategy initially identified 8,392 studies, out of those 42 relevant articles were included in the qualitative and 39 in the quantitative synthesis of this meta-analysis. The study selection process is shown in **Figure 1**. The summary of the characteristics of the studies included in our analysis is shown in **Table 1**. In case of six studies missing data for intention-to-treat analysis were imputed (27, 40, 43, 44, 55, 56).

**Table 1:** Baseline characteristics of trials included.

Author, year/reference no.	Region/country	N° of centers	N° of patients in BP/control group	Age BP/control group (mean years)	Female ratio	Active substance	Dosage (mg)	Control group	Follow-up (months)	Patients with preexisting and/or previous GI diseases excluded	Incidence of nonsevere AE	Incidence of severe AE
Adachi et al., 2009 (18)	Canada and Colombia	34	291/147	65.4/65.7	100%	Alendronate	10	Placebo	3	No	5.5%	0.5%
Ascott-Evans 2003 (19)	Argentina, Australia, Brazil, New Zealand, Africa, Europe	18	95/49	57.3/57.3	100%	Alendronate	10	Placebo	12	No	14.6%	0.0%
Bauer et al., 2000 (20)	United States of America	11	3,236/3,223	68.6/68.7	100%	Alendronate	5	Placebo	45	Yes	54.7%	7.5%
Bell et al., 2002 (21)	United States of America	8	33/32	66.4/65.9	100%	Alendronate	10	Placebo	24	Yes	33.8%	3.1%
Black et al., 1996 (22)	United States of America	11	1,022/1,005	70.7/71	100%	Alendronate	5, after 2 years 10	Placebo	36	Yes	42.8%	5.0%
Bone et al., 2000 (23)	United States of America	18	92/50	61/62	100%	Alendronate	10	Placebo	24	Yes	18.3%	0.0%
Boonen et al., 2009 (24)	Eastern and Western Europe, Lebanon, Australia, USA	24	191/93	60/62	0%	Risedronate	35	Placebo	24	No	10.9%	3.5%
Chesnut et al., 2004 (25)	Canada, United States of America, Europe	73	975/977	69/69	100%	Ibandronate	2.5	Placebo	36	No	20.8%	10.3%
						Ibandronate	20 *				20.9%	9.9%
Clemmesen et al., 1997 (26)	Denmark, Belgium	2	44/44	67/70	100%	Risedronate	2.5	Placebo	36	NI	1.1%	6.8%
				68/70		Risedronate	2.5⁑				3.4%	6.8%
Cryer et al., 2005/1 (27)	United States of America	51	224/230	64.6/65.8	100%	Alendronate	70	Placebo	6	Yes	19.8%	1.3%
Cryer et al., 2005/2 (28)	United States of America	48	224/226	66.6/66.8	92.5%	Alendronate	70	Placebo	3	Yes	12.4%	0.0%
Cummings et al., 1998 (29)	United States of America	11	2,214/2,218	67.6/67.7	100%	Alendronate	5, after 2 years 10	Placebo	24	Yes	23.6%	0.8%
Downs et al., 2000 (30)	United States of America	24	118/58	64.6/64.6	100%	Alendronate	10	Placebo	12	Only esophageal motility disorders	18.8%	0.0%
Eisman et al., 2004 (31)	Europe, Australia, USA. Africa, Asia-Pacific	44	225/224	63.6/63.6	94.2%	Alendronate	70	Placebo	3	No	10.0%	1.1%
Felsenberg et al., 1998 (32)	Argentina, Australia, Canada, Colombia, Europe	62	219/223	64.1/63.3	100%	Alendronate	10	Placebo	12	Yes	29.2%	2.5%
Fogelman et al., 2000 (33)	UK, France, Netherlands, Belgium, Germany	13	184/180	65/64	100%	Risedronate	2.5	Placebo	24	No	23.4%	6.0%
			177/180			Risedronate	5				21.8%	5.6%
Greenspan et al., 2002 (34)	United States of America	48	224/226	66.6/66.8	92.4%	Alendronate	70	Placebo	3	Only esophageal motility disorders	14.0%	1.3%
Harris et al., 1999 (35)	North America	110	813/815	69/68	100%	Risedronate	5	Placebo	36	No	24.4%	5.7%
Hosking et al., 2003 (36)	Europe and Brazil	38	222/108	68.9/69.6	100%	Risedronate	5	Placebo	12	Only esophageal motility disorders	0.3%	1.2%
			219/108	69,2/69,6		Alendronate	70				0.6%	0.9%
Ilter et al., 2006 (37)	Turkey	1	41/41	56.4/56.2	100%	Risedronate	5	Calcium+ Vitamin D	3	No	17.1%	1.2%
			41/41	55,9/56,2		Risedronate	35				15.9%	0.0%
Iwamoto et al., 2001 (38)	Japan	1	25/24	64.3/66	100%	Etidronate	200^§^	Calcium lactate	24	No	0.0%	0.0%
Johnell et al., 2002 (39)	Australia, Belgium, Canada, Italy, Mexico, South Africa, Spain, Sweden	30	83/82	63.7/63.8	100%	Alendronate	10	Placebo	10	Yes	8.5%	0.0%
Kung et al., 2000 (40)	China	1	35/35	64/65	100%	Alendronate	10	Placebo	12	Yes	17.1%	2.9%
Kushida et al., 2004 (41)	Japan	55	90/80	71.2/72.6	100%	Alendronate	5	Alfacalcidol	36	Yes	8.2%	4.7%
Lanza et al., 2002 (42)	United States of America	5	126/126	54.7/54.7	ND	Alendronate	70	Placebo	2.5	Yes	22.2%	0.4%
Lanza et al., 2000 (43)	United States of America	4	90/36	54.3/53.5	63.5%	Alendronate	40	Placebo	1	Yes	ND	7.1%
			89/36		63.2%	Risedronate	30				ND	8.8%
Lau et al., 2000 (44)	China	1	53/47	74/74	100%	Alendronate	10	Placebo	12	Yes	12.0%	0.0%
Leung et al., 2005 (45)	China	4	31/34	67/67	100%	Risedronate	5	Placebo	12	Yes	3.1%	0.0%
Liberman et al., 1995 (46)	USA, Canada, Australia, Europe, Israel, New Zealand, Mexico, South America	28	175/355	64/64	100%	Alendronate	5	Placebo	36	Yes	0.0%	ND
			175/355			Alendronate	10				17.2%	ND
			175/355			Alendronate	20				0.0%	ND
McClung et al., 2001 (47)	North America, Europe, New Zealand, Australia	183	3,093/3,134	ND	100%	Risedronate	2.5	Placebo	36	No	17.0%	2.1%
			3,104/3,134			Risedronate	5				16.8%	2.2%
Miller et al., 2000 (48)	United States of America	38	88/84	67/67.1	100%	Alendronate	10	Placebo	2	No	14.0%	2.3%
Murphy et al., 2001 (49)	United States of America	10	109/36	72.9/70.9	100%	Alendronate	10	Placebo	18	Yes	2.8%	1.4%
Orwoll et al., 2000 (50)	United States of America	20	146/95	63/63	0%	Alendronate	10	Placebo	24	Yes	15.8%	0.8%
Pols et al., 1999 (51)	Europe, Canada, Latin America, Australia, South Africa, China	153	950/958	62.8/62.8	100%	Alendronate	10	Placebo	12	Yes	20.8%	3.7%
Reginster et al., 2000 (52)	Europe, Australia	80	408/407	71/71	100%	Risedronate	2.5	Placebo	24	No	18.4%	8.0%
			407/407			Risedronate	5		36		19.9%	7.6%
Ryan et al., 2000 (53)	United Kingdom	2	41/41	65.6/66.1	90.1%	Pamidronate	150^†^	Placebo	24	Yes	54.3%	0.0%
			40/41	63.8/61.1		Pamidronate	300^‡^				36.6%	0.0%
Seeman et al., 2010 (54)	Argentina, Australia, Canada, France, USA	9	81/83	60.7/60.8	100%	Alendronate	70	Placebo	12	No	54.9%	0.0%
Shiraki et al., 1999 (55)	Japan	63	102/100	63.53/63.14	100%	Alendronate	5	Alfacalcidol	12	No	19.3%	0.5%
Shiraki et al., 2003 (56)	Japan	70	52/54	60,7/60,5	99%	Risedronate	1	Placebo	3		6.1%	0.0%
			49/54	60,6/60,5		Risedronate	2.5			No	14.6%	0.0%
			56/54	60,2/60,5		Risedronate	5				14.4%	0.0%
Tucci et al., 1996 (57)	United States of America	18	98/192	66.5/64.2	100%	Alendronate	5	Placebo	18		12.4%	1.4%
			94/192	63,9/64,2		Alendronate	10			Yes	14.0%	0.7%
			94/192	63,8/64,2		Alendronate	20				13.3%	1.4%
Yan et al., 2009 (58)	China	7	280/280	65.19/64.66	100%	Alendronate	70	Placebo	12	No	16.1%	0.0%
You et al., 2011 (59)	China	1	90/90	62.71/61.93	100%	Alendronate	70⁑	Colecalciferol	12	No	ND	ND

All trials were randomized, controlled multicenter studies, except Ilter, Iwamoto, Lau, You, which are single-center studies. Ten milligrams of Alendronate was taken in a daily regime, and 70 mg was taken once per week. * cyclic intermittent therapy: 20 mg ibadronate every other day for 2 weeks, every 3 months. cyclic intermittent therapy: 2.5 mg risedronate daily for 2 weeks, every 3 months. §Cyclic intermittent therapy: 200 mg etidronate daily for 2 weeks, every 3 months. †Cyclic intermittent therapy: 150 mg pamidronate for 4 weeks, followed by 4 weeks of placebo. ‡Cyclic intermittent therapy: 300 mg pamidronate for 4 weeks, followed by 12 weeks of placebo. ⁑ 70 mg of alendronate every 2 weeks. BP, bisphosphonate; ND, no data; NI, no information.

### Adverse Events

The forty-two RCTs included 39,047 patients with 9,999 non-severe and 1,503 severe GI adverse events. The incidence of non-severe and severe adverse events ranged between 0.3–54.9, and 0–10.3%, respectively. The most common non-severe adverse events were nausea, vomiting, dyspepsia, and abdominal pain, while the vast majority of the severe side effects occurred in the esophagus (**Supplementary Table 2**).

### Intervention: Bisphosphonates

Our meta-analysis included data from studies with four bisphosphonates: alendronate, risedronate, etidronate, and ibandronate. One study with pamidronate was included in the qualitative synthesis. All studies used orally administered bisphosphonates. Dosages and other details are shown in **Table 1**.

### Results of Statistical Analysis

#### Bisphosphonate Use Is Not Associated With the Risk of Non-Severe Adverse Events

**Figure 2:**
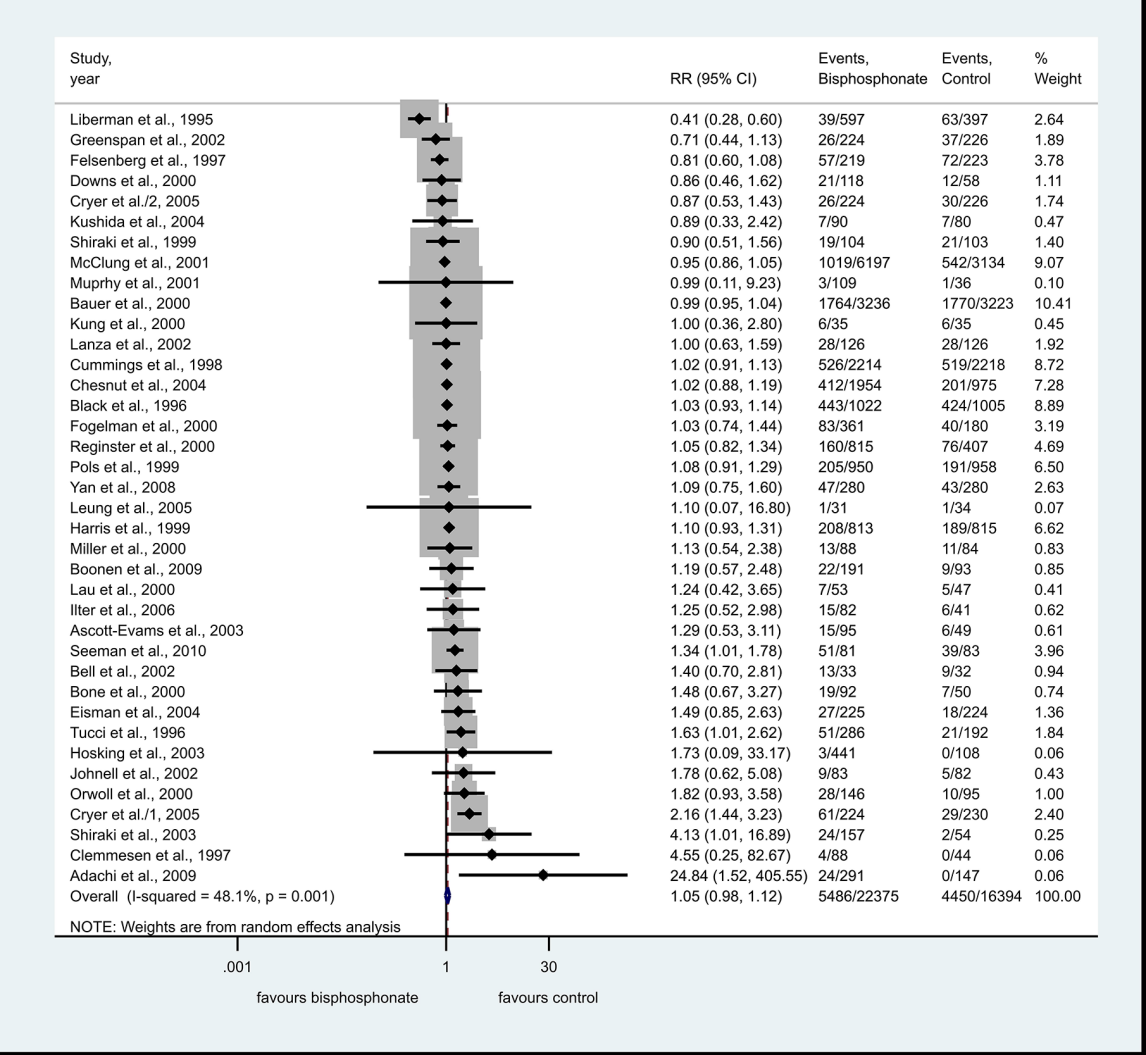
Forest plot of non-severe adverse events.

The analysis for non-severe GI adverse events included 39 studies in the quantitative analysis. The number of overall non-severe GI adverse events were 5,486 in the bisphosphonate group and 4,450 in the control group. Compared against controls, the bisphosphonate use was not associated with the risk of non-severe side effects, RR=1.05, CI: 0.98–1.12, p=0.207 the heterogeneity was moderate: I^2^ = 48.1%, p=0.001 (**Figure 2**).

Among non-serious GI adverse events alendronate, risedronate and ibandronate had 27, ten, and one studies included, respectively. Subgroup analysis for the three different bisphosphonates did not show an association with the risk of non-severe side effects (**Supplementary Figure 1**).

#### Bisphosphonate Use Is Not Associated With Increased Risk of Severe Adverse Events

The number of overall severe GI adverse events were 874 in the bisphosphonate group and 629 in the control group.

**Figure 3:**
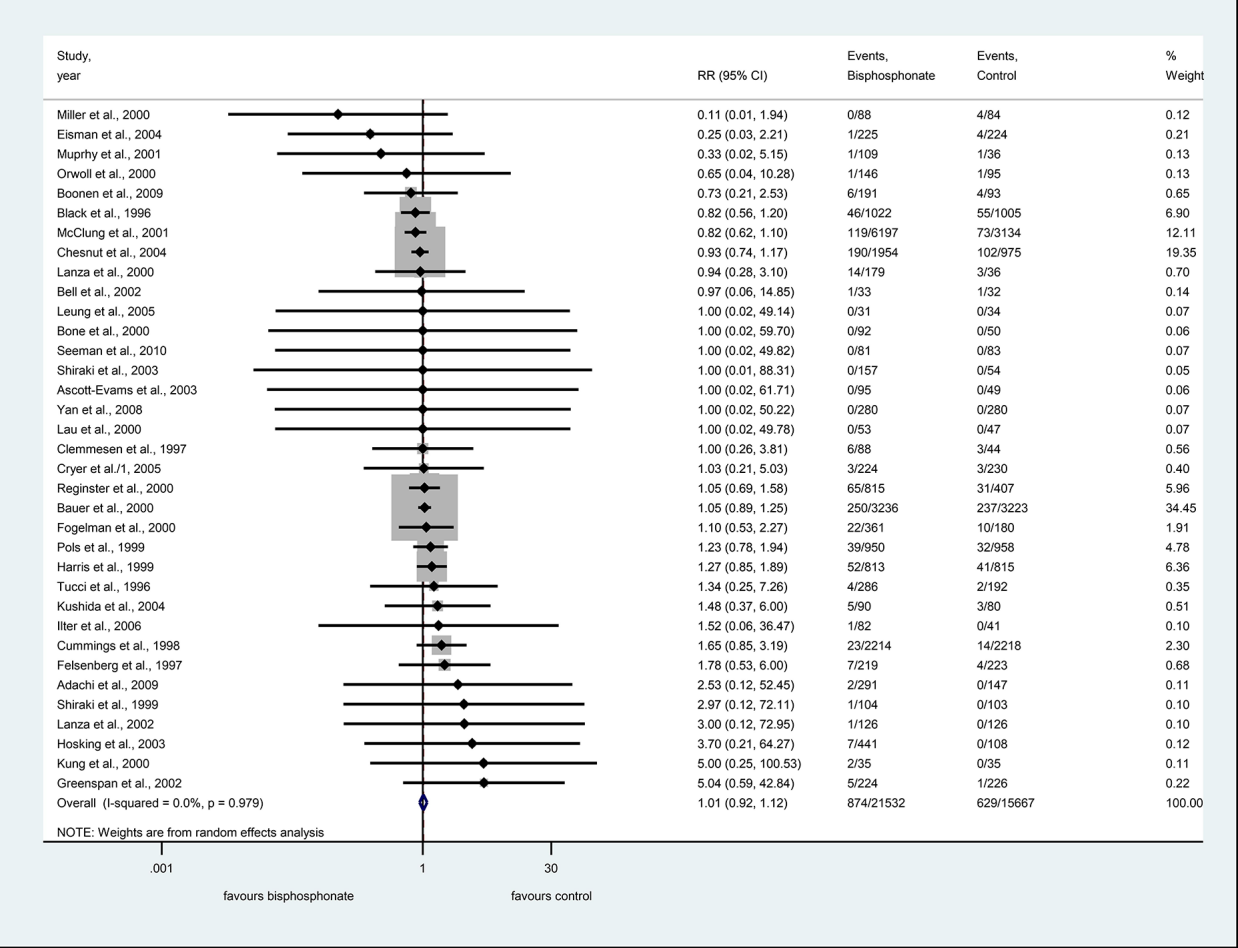
Forest plot of severe adverse events.

The bisphosphonate use was not associated with the risk of severe side effects, compared against controls, RR=1.01, CI: 0.92–1.12, p=0.776; there was no significant heterogeneity: I^2^ = 0.0%, p=0.979 (**Figure 3**).

Among serious upper GI events alendronate, risedronate, ibandronate, and etidronate had 24, ten, one, and one studies included, respectively. Subgroup analysis for the three different bisphosphonates did not show an association with the risk of non-severe side effects (**Supplementary Figure 2**).

#### Subgroup Analysis of Trials With the Primary Outcome of GI Tolerability of BP Therapy Showed No Increased Risk of GI Adverse Events

**Figure 4:**
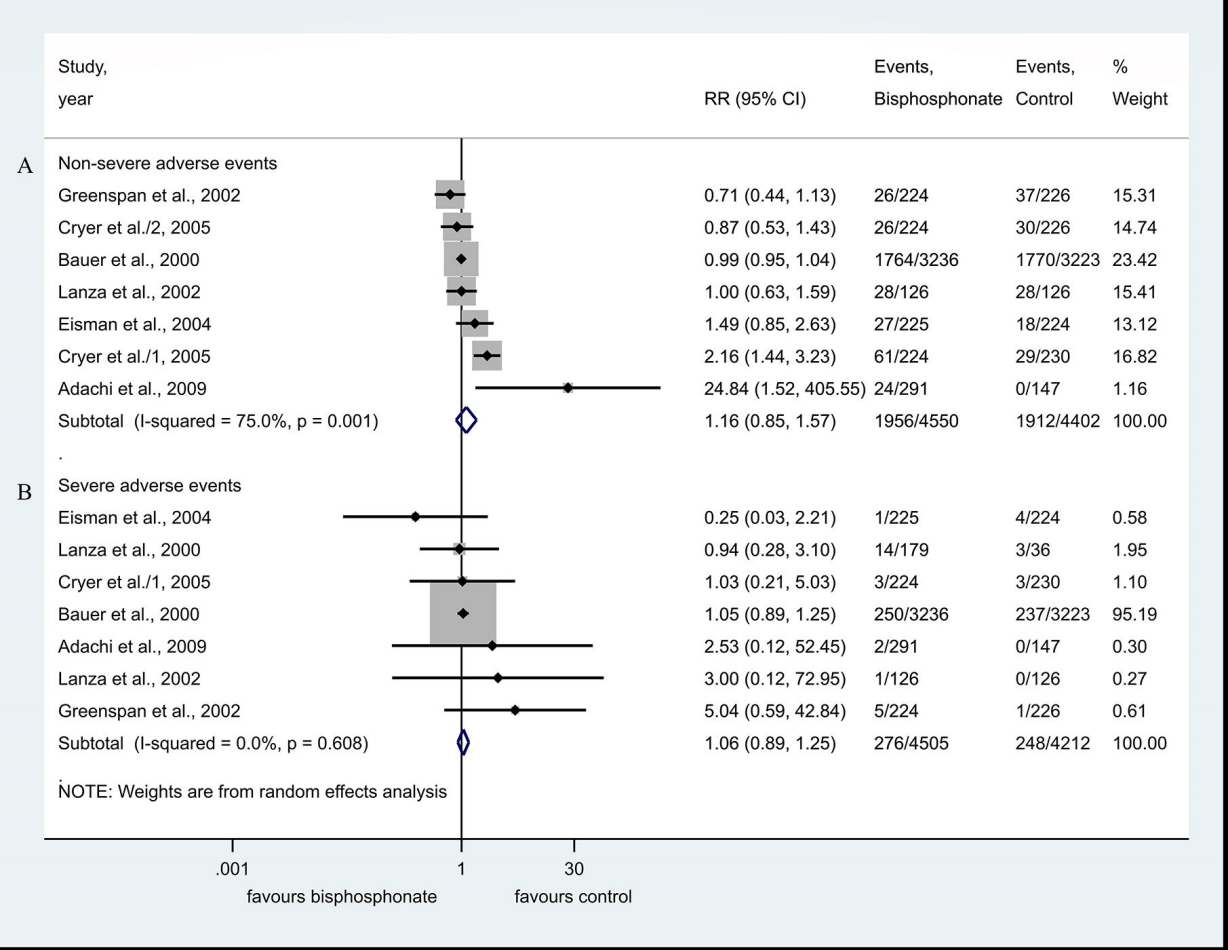
**(A, B)** Subgroup analysis of trials with the primary outcome of GI tolerability of BP therapy.

The number of overall non-severe GI adverse events was 1,956 in the bisphosphonate group and 1,912 in the control group. Compared to controls, the BP use was not associated with the risk of non-severe side effects, RR=1.16, CI: 0.85–1.57, p=0.356, with considerable heterogeneity: I^2^ = 75.0%, p=0.001 (**Figure 4A**).

The number of overall severe GI adverse events was 276 in the bisphosphonate group and 248 in the control group. The BP use was not associated with the risk of severe side effects, compared against controls, RR=1.06, CI: 0.89–1.25, p=0.529; there was no significant heterogeneity: I^2^ = 0.0%, p=0.608 (**Figure 4B**).

#### Long-Term Administration of Bisphosphonate Is Not Associated With Increased Risk of Side Effects

In 15 eligible articles, there was no association between BP use and the risk of non-severe side effects in the subgroup of studies, where the treatment was at least 24 months, RR=1.00, CI: 0.93–1.08, p=0.983, heterogeneity was moderate: I^2^ = 53.9%, p=0.007 (**Supplementary Figure 3**).

In 14 eligible articles, there was no association with the risk of severe side effects in the subgroup of studies where the treatment was at least 24 months, RR=1.00, CI: 0.90–1.11, p=0.944 there was no significant heterogeneity: I^2^ = 0.0%, p=0.858 (**Supplementary Figure 4**).

#### Non-Severe and Severe Adverse Events in the Context of the Most Commonly Used BP Therapies

Subgroup analysis of the most commonly used BP, alendronate 10 mg/day or once-weekly alendronate 70 mg, revealed an increased risk of non-severe GI adverse events compared against controls, RR=1.16, CI: 1.00–1.36, p=0.056, with moderate heterogeneity: I^2^ = 40.7%, p=0.031, while the risk of severe adverse events was not increased in this subgroup RR=1.20, CI: 0.83–1.74, p=0.328, without significant heterogeneity: I^2^ = 0.0%, p=0.897 (**Supplementary Figures 5**, **6**).

#### Trial Sequential Analysis

In case of non-severe adverse events, the cumulative z-curve crosses the futility boundary, which provided evidence indicating that no significant difference exists between the groups, and thus, further trials are not required (**Supplementary Figure 7**). In case of severe adverse events, the same conclusion could not be drawn as the acquired information size was substantially below the required information size (0.76%) by performing the TSA.

#### Risk of Bias Assessment

According to the Revised Cochrane Risk of Bias Assessment tool for RCTs the risk of bias was low in 25 studies, there were some concerns in 11, and high in six studies. Nearly all studies carried an unknown risk of reporting bias due to the lack of pre-study protocols. The detailed results of the assessment are shown in **Supplementary Table 2**.

#### Publication Bias

In case of non-severe side effects, both the visual assessment of the funnel plot and the Egger’s test, p=0.046, revealed small study effect, so the presence of publication bias was strongly suspected (**Supplementary Figure 8**). Therefore, the metanalytical pooled estimation was repeated by the use of trim and fill method, which did not change the overall risk association (RR= 0.99, CI: 0.91 – 1.07).

In case of severe side effects, publication bias was undetected by visual inspection of the funnel plot and Egger’s test p = 0.307 (**Supplementary Figure 9**).

#### Grade of Evidence

For non-severe GI side effects, the evidence was graded as very low due to inconsistency, indirectness, and publication bias and for severe GI side effects, the evidence was graded moderate due to indirectness (**Table 2**).

**Table 2:** Grade of evidence.

Summary of findings:						
Gastrointestinal adverse events of bisphosphonates compared to Control						
**Patient or population:** Adult patients with osteoporosis**Intervention:** Bisphosphonate**Comparison:** Control						
**Outcomes**	Anticipated absolute effects^*^ (95% CI)		**Relative effect** **(95% CI)**	**№ of participants** **(studies)**	**Certainty of the evidence** **(GRADE)**	**Importance**
	**Risk with Control**	**Risk with Bisphosphonate**				
Non-severe GI adverse events	271 per 1,000	285 per 1,000 (266 to 304)	RR 1.05 (0.98 to 1.12)	38,769 (38 RCTs)	⨁◯◯◯ VERY LOW ^a,b,c^	IMPORTANT
Severe GI adverse events	40 per 1,000	41 per 1,000 (37 to 45)	RR 1.01 (0.92 to 1.12)	37199 (35 RCTs)	⨁⨁⨁◯ MODERATE ^b^	CRITICAL
*The risk in the intervention group (and its 95% confidence interval) is based on the assumed risk in the comparison group and the **relative effect** of the intervention (and its 95% CI). **CI,** confidence interval; **RR**, risk ratio						
**GRADE Working Group grades of evidence** **High certainty:** We are very confident that the true effect lies close to that of the estimate of the effect **Moderate certainty:** We are moderately confident in the effect estimate: The true effect is likely to be close to the estimate of the effect, but there is a possibility that it is substantially different **Low certainty:** Our confidence in the effect estimate is limited: The true effect may be substantially different from the estimate of the effect **Very low certainty:** We have very little confidence in the effect estimate: The true effect is likely to be substantially different from the estimate of effect						

^a^Heterogeneity was moderate among the studies included in the analysis of the risk of non-severe GI adverse events (I^2^ = 48.1%, p = 0.001)

^b^There were major differences in the intervention groups of the studies included regarding the used drug (alendronate/ibandronate/risedronate/pamidronate), dosage, and administration intervals.

^c^The funnel plot of this outcome revealed asymmetry and Eger’s test suggested small study effect (p = 0.046).

## Discussion

The pathophysiology of the bisphosphonate induced esophageal and gastric erosions has not been elucidated. In vitro studies suggest that the mucosal damage is produced through topical irritant effects on the gastric epithelium (60, 61). It is also described that BPs are competitively displacing the phospholipids from the mucus gel layer, therefore the mucosal hydrophobic barrier is attenuated and mucosal healing is hindered (62–64).

Our results from 42 RCTs with nearly 40,000 participants showed that the incidence of non-severe and severe GI side effects ranged between 0–54.9 and 0–10.3%, respectively. Neither the risk of non-severe nor the risk of severe adverse GI events was associated with the oral bisphosphonate use in osteoporotic patients.

Our meta-analysis is the first that objectified the risk of non-severe and severe GI side effects separately. When the use of bisphosphonates became widespread, it was predicted that gastroenterologists would see more patients with consequent GI problems (65).

Two previous reviews assessed the risk of GI side effects of risedronate and ibandronate separately (66, 67). A meta-analysis of nine RCTs focused on the GI tolerability of alendronate (68). A comprehensive network meta-analysis compared the GI safety of BPs, but they did not calculate the risk of side effects of bisphosphonates against placebo (69). The assessment of the risk of severe GI side effects was not based on the true severity of the GI side effects but how they were classified in the original studies.

### Studies With a Primary Outcome of Gastrointestinal Adverse Events

Eight out of the included RCTs had GI adverse events as the primary outcome (18, 27, 43–42). Only two of these studies proved that the risk of non-severe GI side effects increased in patients taking BPs (18, 27). None of them showed an association between BPs and severe GI side effects.

While the first trial of Cryer et al. managed to detect an increased risk of non-severe GI side effects, their second trial, in which approximately half of participants took non-steroidal anti-inflammatory drugs on both arms, could not demonstrate this association (27, 28).

A study in 2,000 assessed each participants’ GI side effects through endoscopic inspection of the mucosa at baseline and completion of the study. They concluded that mucosal damage did not translate into clinically significant symptoms or side effects (43).

Miller et al. investigated whether previous GI side effects of BP therapy predisposed to recurrent side effects after rechallenge with alendronate. They found no significant risk of severe or non-severe GI side effects associated with the alendronate use. The incidence of non-severe and severe GI side effects were 14 and 2.3%, respectively (48).

Since the introduction of the BPs in the treatment of osteoporosis multiple studies confirmed their GI tolerability. Even if they have non-severe GI side effects, their use rarely results in severe complications needing the attention of the gastroenterologist.

Oral BPs are nowadays recommended to take with water and to avoid lying down after intake to avoid esophageal irritation. These precautions might reduce the incidence of upper GI side-effects in more recent RCTs and in current clinical practice.

### Strength of Our Study

Our work, which includes a large number of RCT-s and participants was conducted following a rigorous methodology. Furthermore, most of the included RCT-s are multinational and multicentric. To date, this is the first meta-analysis which quantified the risk of non-severe and severe GI side effects of oral BP therapy.

### Limitations of Our Study

In most of the studies, GI side effects were a secondary outcome and were not powered statistically to reveal a significant difference in that respect. The heterogeneity of the strategy of vigilance for side effects probably explains the wide range of incidence of side effects; however, it did not translate to statistical heterogeneity among the severe side effects. The differences between sexes, ages, length of the studies and various definitions in addition to different approaches of the screening of non-severe side effects resulted in moderate and significant heterogeneity among the studies. Also, the included studies likely used different sets of predetermined GI side effects during the screening for side effects. Another significant limitation of the study is that in 24 of 42 RCTs included in the analysis, pre-existing and/or previous GI diseases were exclusion criteria. The conclusions drawn from the meta-analysis are therefore restricted to selected populations, and the results must be interpreted with caution. These considerations are reflected in **Table 2**, in which the grades of evidence were rated very low for non-severe GI side-effects and only moderate for severe GI side-effects.

Risk assessment revealed unclear bias in most of the studies concerning the reporting of the results.

## Conclusion

### Implications for Research

Although the results suggest that bisphosphonates do not increase the risk of GI side effects in the general osteoporotic population, we cannot conclude whether they are safe to use in a high-risk population with preexisting GI pathologies (e.g. gastroesophageal reflux, peptic ulcer disease, etc.). Therefore future phase III. trials should focus on these high-risk populations.

### Implications for Practice

Bisphosphonates seem to be safe in the osteoporotic population concerning the GI side effects, however other factors need to be considered when decisions on treatment are made.

## Data Availability Statement

The original contributions presented in the study are included in the article/**Supplementary Material**. Further inquiries can be directed to the corresponding author.

## Author Contributions

ZD, BE, and PH conceived the study. ZD, NV, and SK wrote the protocol. ZD, EC, and LS did the literature search. ZD and NV screened the records and extracted data. LS and AP validated the extracted data. ZD, NV, and EC assessed the quality of the included studies. LH did the statistical analysis. ZD and NV prepared the tables. BE, ZD, and NV wrote the first draft of this manuscript. PH, SK, and AP supervised the manuscript and approved the submitted draft. BE is the guarantor of this paper. All authors contributed to the article and approved the submitted version.

## Funding

Sponsors were not involved in the design, data collection, analysis, interpretation, or preparation of the manuscript. Financial support: Supported by the Economic Development and Innovation Operative Programme Grant (GINOP-2.3.2-15-2016-00048) and the Human Resources Development Operational Programme Grants (EFOP-3.6.2-16-2017-00006).

## Conflict of Interest

The authors declare that the research was conducted in the absence of any commercial or financial relationships that could be construed as a potential conflict of interest.

## Supplementary Material

Supplementary Figure 1 Non-severe adverse events subgroup by active substance.

Supplementary Figure 2 Severe adverse events subgroup by active substance.

Supplementary Figure 3 Non-severe GI side effects subgroup by more than 24 months of treatment.

Supplementary Figure 4 Severe GI side effects subgroup by more than 24 months of treatment.

Supplementary Figure 5 Non-severe adverse events in the context of the most commonly used BP therapies: 70 mg/week and 10 mg/day alendronate per os.

Supplementary Figure 6 Severe adverse events in the context of the most commonly used BP therapies: 70 mg/week and 10 mg/day alendronate per os.

Supplementary Figure 7 Trial Sequential Analysis of non-severe GI. adverse events

Supplementary Figure 8 Funnel plot of non-severe GI adverse events.

Supplementary Figure 9 Funnel plot of severe GI adverse events.

Supplementary Table 1 FDA criteria of non-severe and severe GI adverse events.

Supplementary Table 2 Risk of Bias Assessment.
