# Supplementary figures and images for: Oral Treatment With Bisphosphonates of Osteoporosis Does Not Increase the Risk of Severe Gastrointestinal Side Effects: A Meta-Analysis of Randomized Controlled Trials
Source: Front Endocrinol (Lausanne). 2020 Nov 10;11:573976. doi: 10.3389/fendo.2020.573976 (PMC7683730; doi:10.3389/fendo.2020.573976)

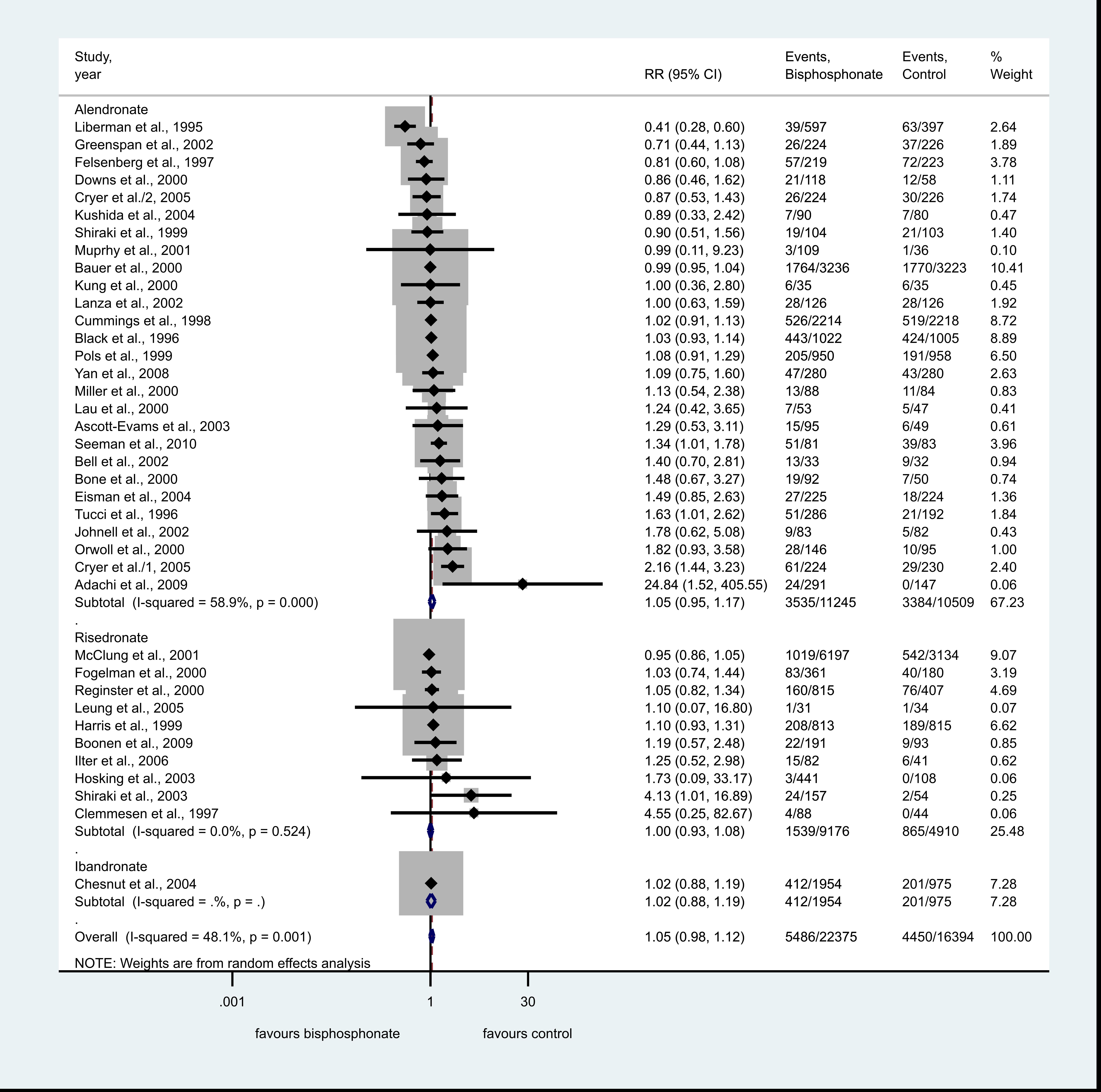

Supplement: Supplementary Figure 1 — Non-severe adverse events subgroup by active substance. [file Image_1.jpeg]

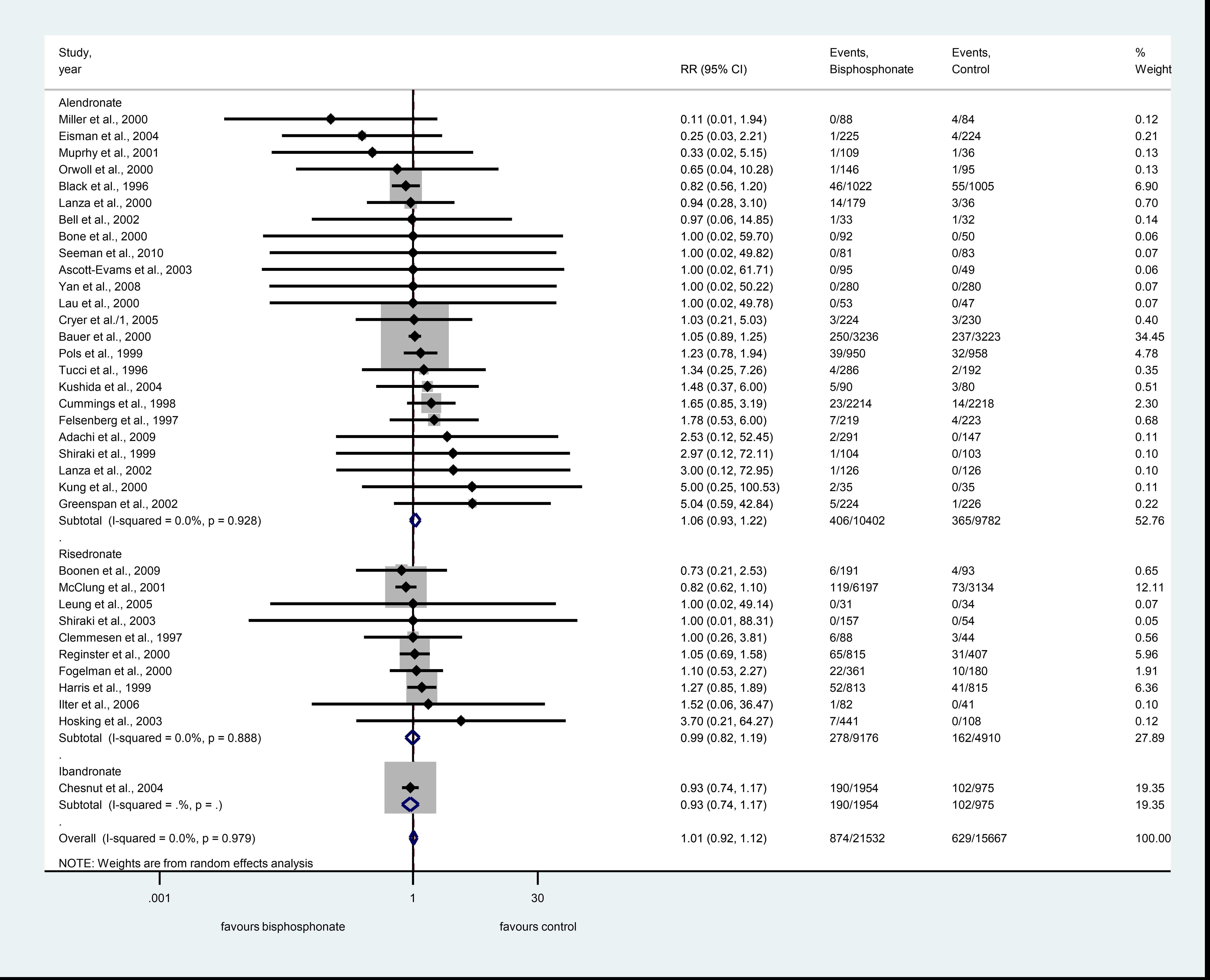

Supplement: Supplementary Figure 2 — Severe adverse events subgroup by active substance. [file Image_2.jpeg]

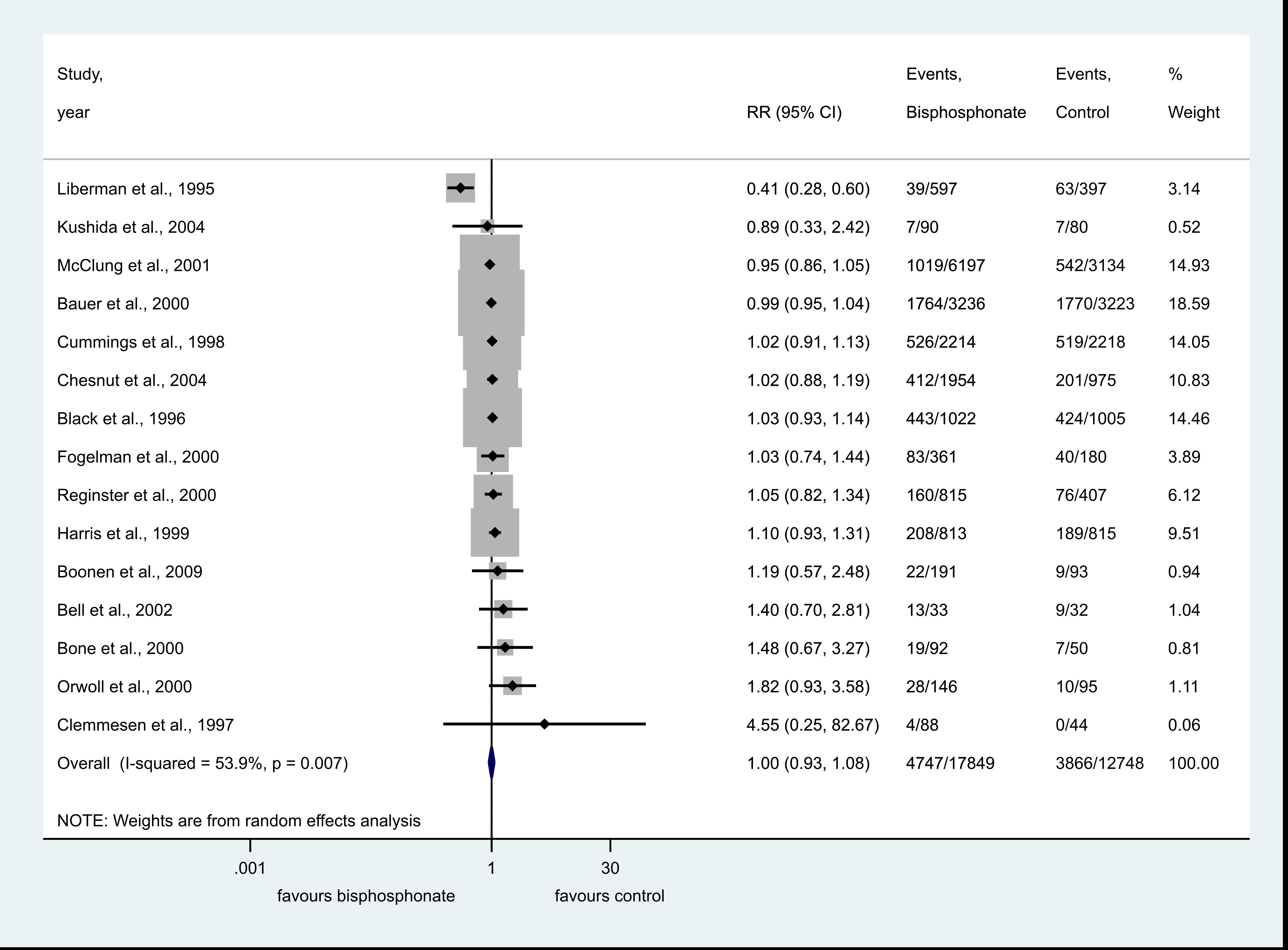

Supplement: Supplementary Figure 3 — Non-severe GI side effects subgroup by more than 24 months of treatment. [file Image_3.jpeg]

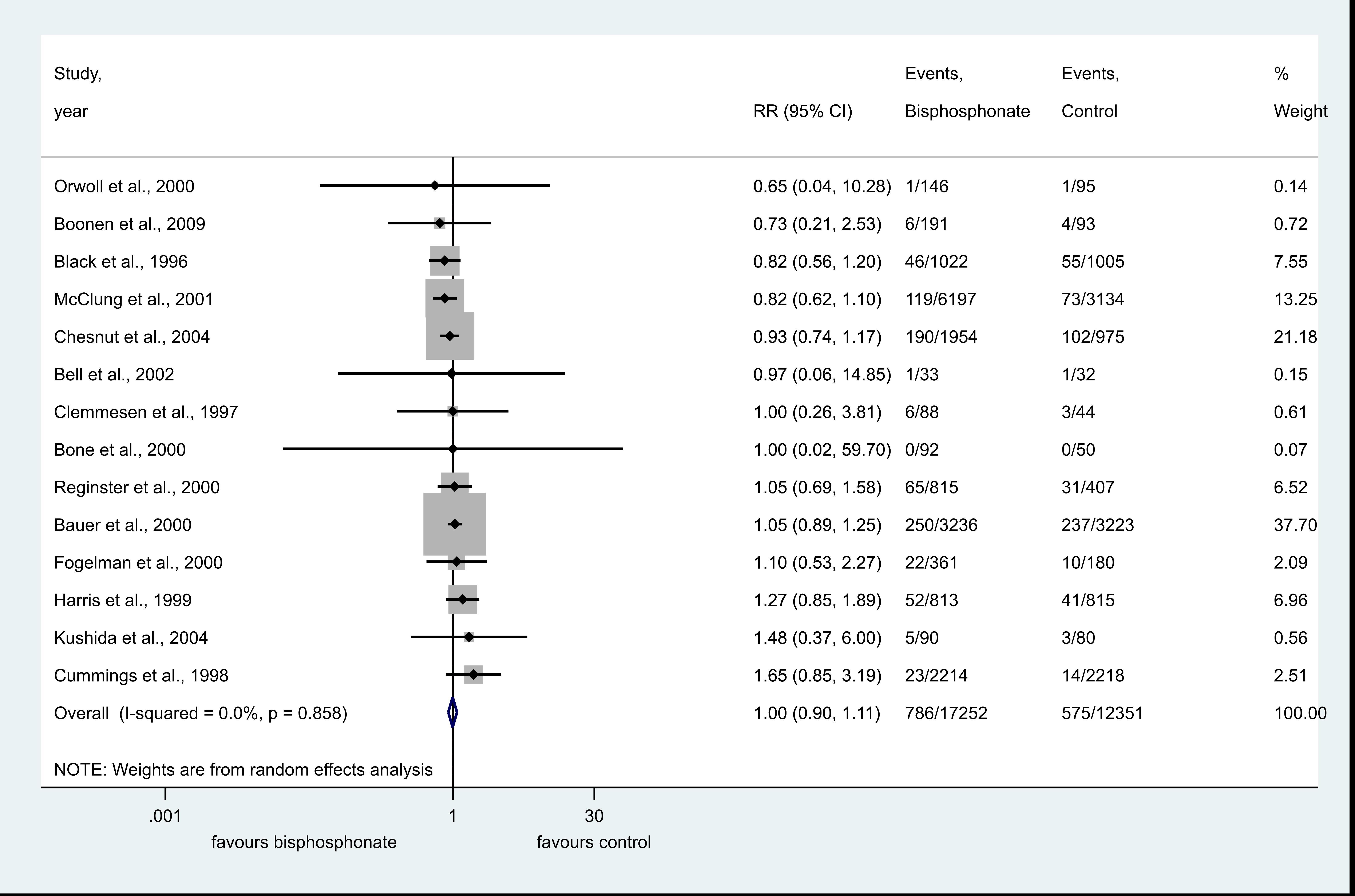

Supplement: Supplementary Figure 4 — Severe GI side effects subgroup by more than 24 months of treatment. [file Image_4.jpeg]

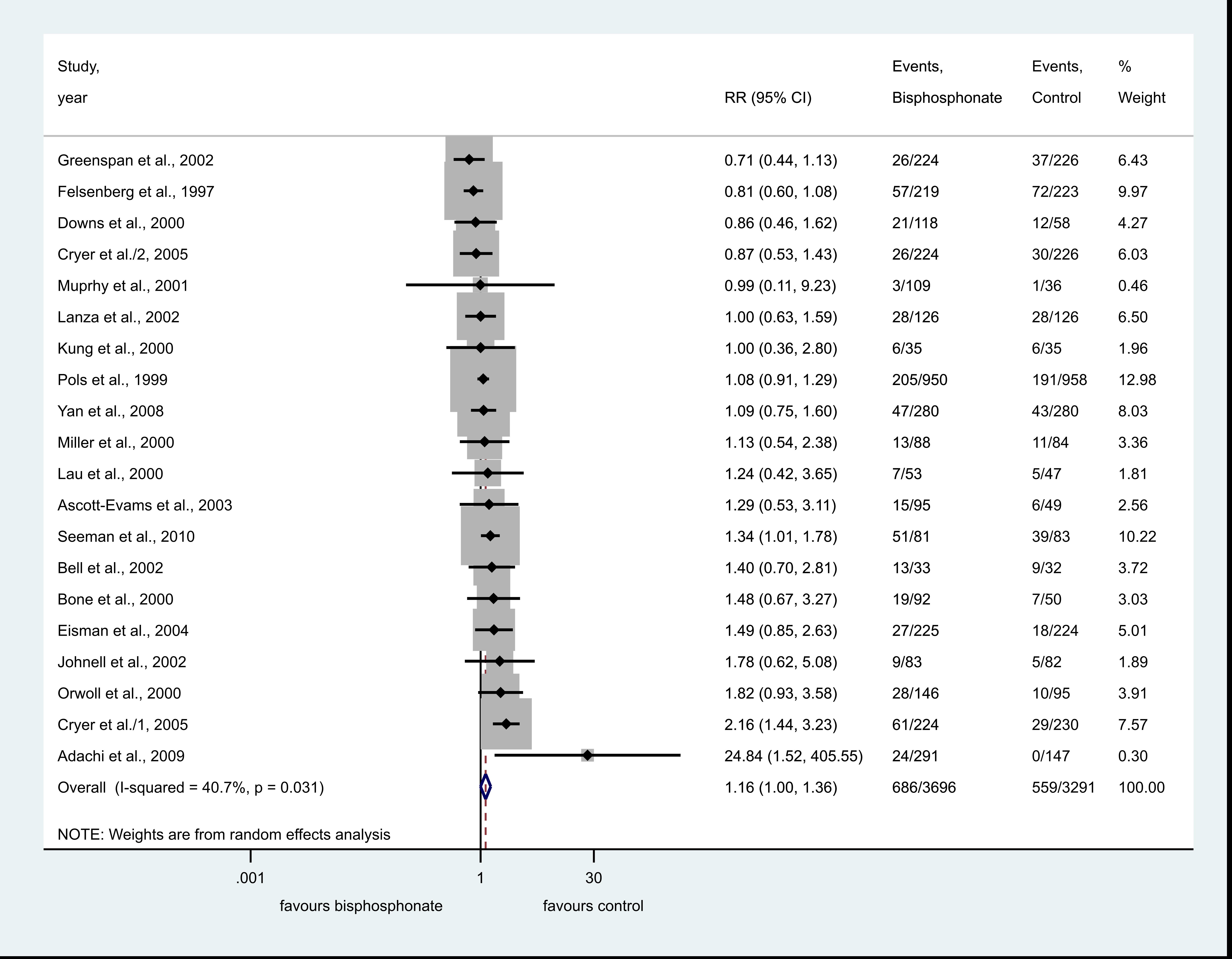

Supplement: Supplementary Figure 5 — Non-severe adverse events in the context of the most commonly used BP therapies: 70 mg/week and 10 mg/day alendronate per os. [file Image_5.jpeg]

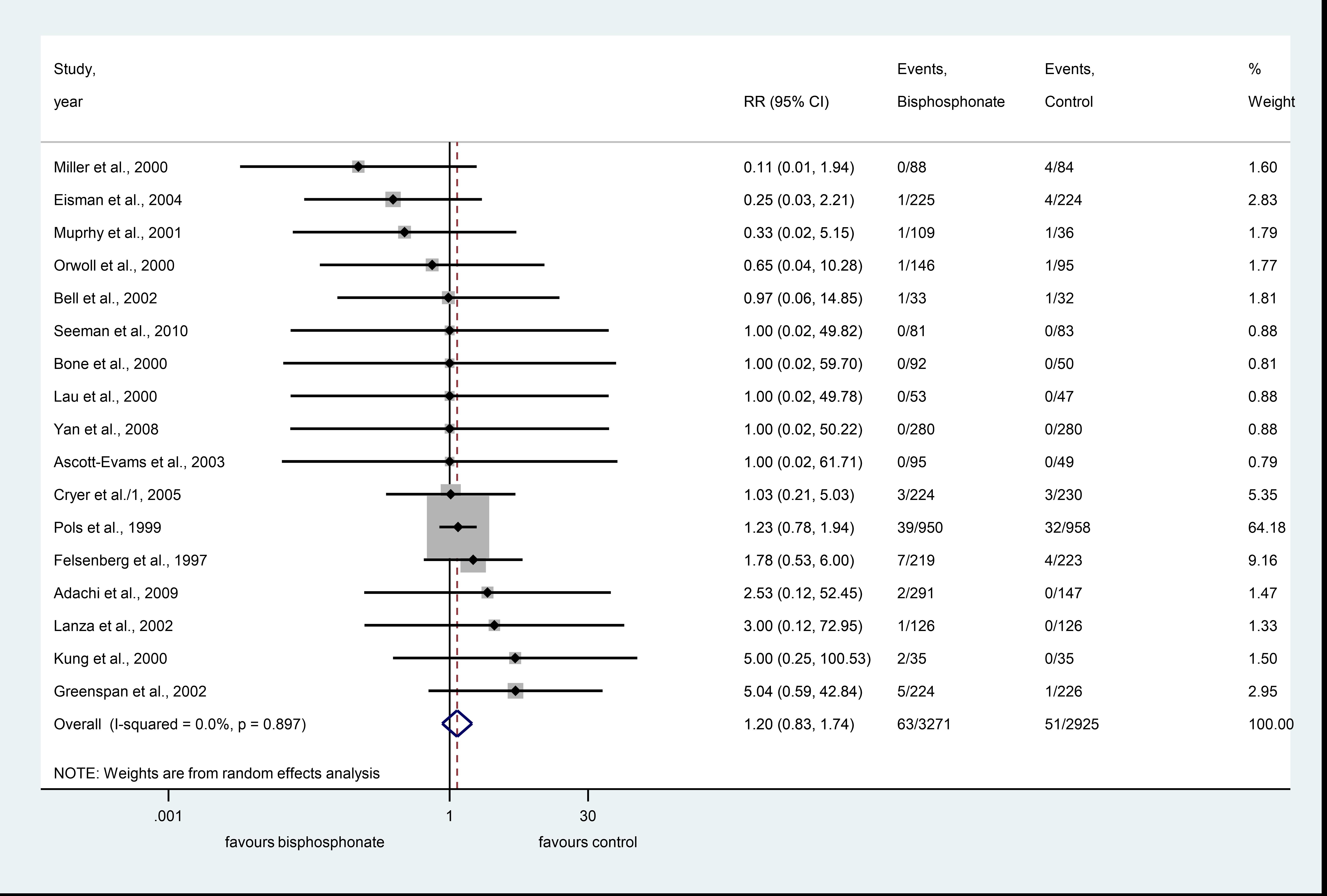

Supplement: Supplementary Figure 6 — Severe adverse events in the context of the most commonly used BP therapies: 70 mg/week and 10 mg/day alendronate per os. [file Image_6.jpeg]

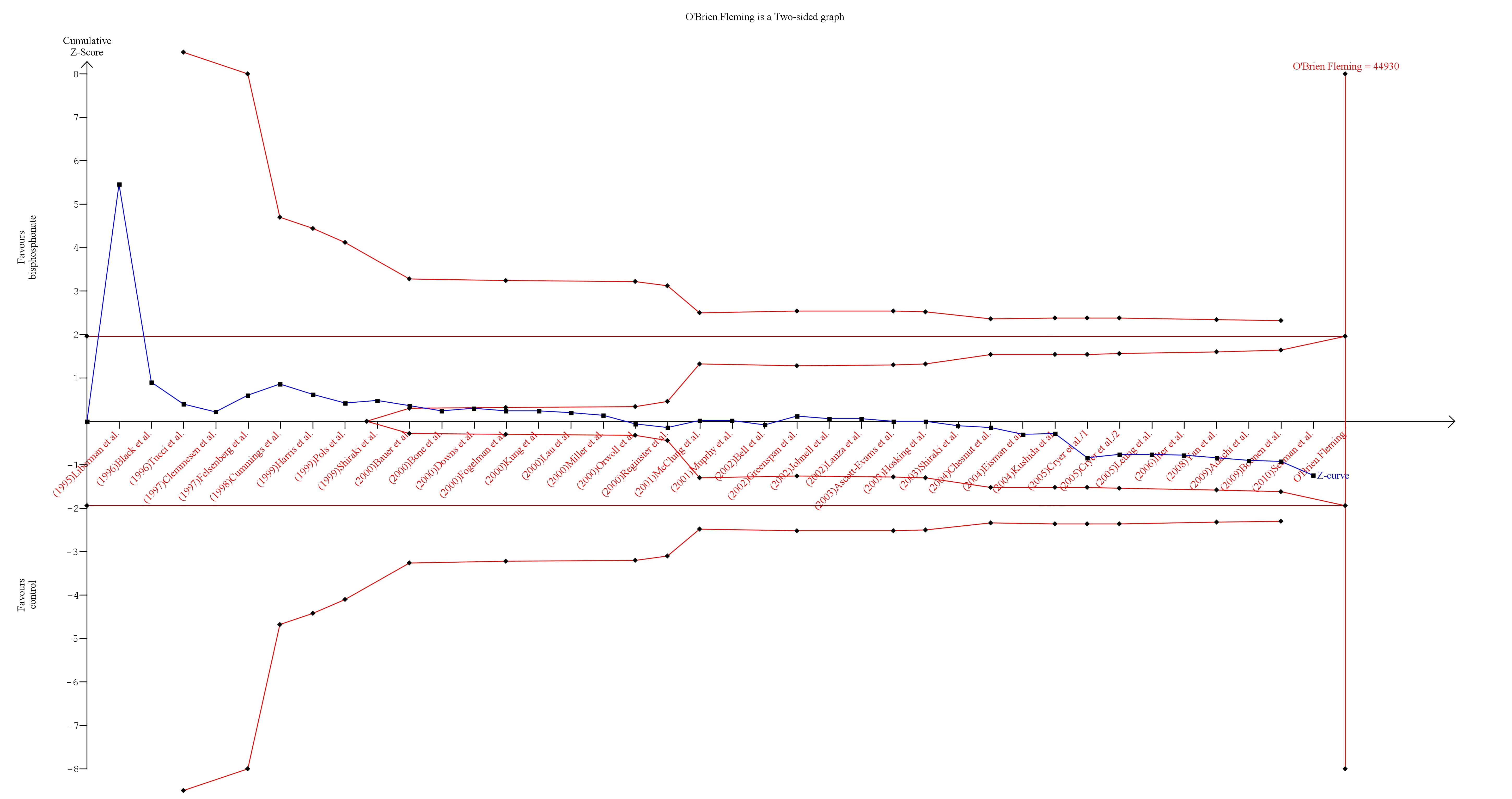

Supplement: Supplementary Figure 7 — Trial Sequential Analysis of non-severe GI. adverse events [file Image_7.jpeg]

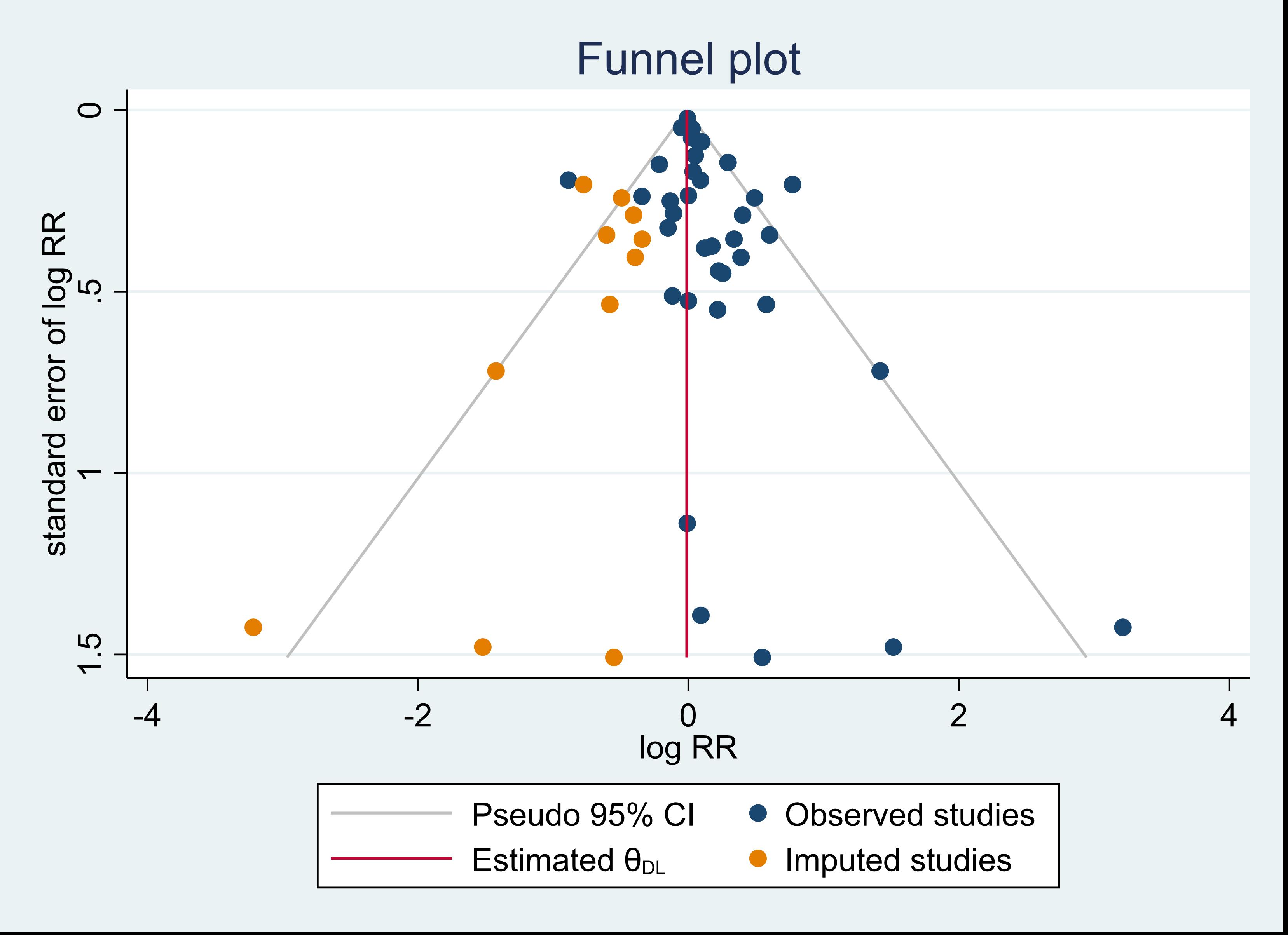

Supplement: Supplementary Figure 8 — Funnel plot of non-severe GI adverse events. [file Image_8.jpeg]

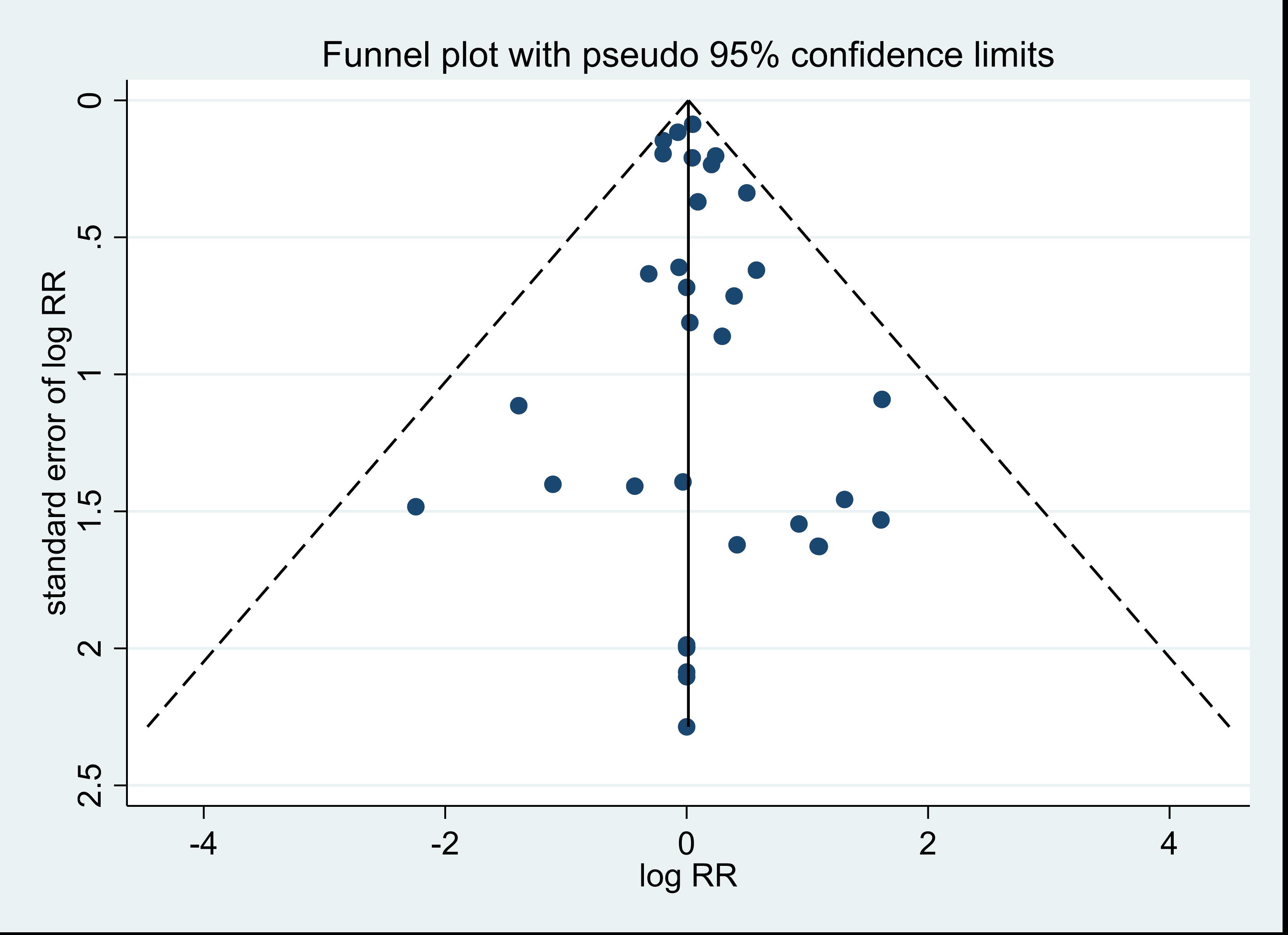

Supplement: Supplementary Figure 9 — Funnel plot of severe GI adverse events. [file Image_9.jpeg]
